# Symmetry dual functional pyrimidine-BODIPY probes for imaging targeting and activity study

**DOI:** 10.3389/fchem.2022.977008

**Published:** 2022-09-20

**Authors:** Shuping Xu, Ying Liu, Zhou Wang, Aolin He, Guofan Jin

**Affiliations:** ^1^ The People’s Hospital of Danyang, Affiliated Danyang Hospital of Nantong University, Zhenjiang, China; ^2^ School of Pharmacy, Jiangsu University, Zhenjiang, China; ^3^ College of Vanadium and Titanium, Panzhihua University, Panzhihua, China; ^4^ Affiliated Kunshan Hospital, Jiangsu University, Suzhou, China

**Keywords:** fluorescence probe, anticancer agent, biological imaging, biological activity, symmetry dual function

## Abstract

Nondestructive diagnosis of tumor has always been the goal of scientists. Fluorescent dyes have become the rising star in the field of cancer diagnosis because of their excellent characteristics. Therefore, in this work, fluorescence probes **d-Y-B** and **dO-Y-B** with anti-tumor activity were constructed by introducing pyrimidine groups with high anti-tumor activity using fluorescence dye BODIPY as parent nucleus. The modified BODIPY group in the structure had the advantage of fluorescent dye, ensuring the strong fluorescence and photosensitivity of the target compound. That ethylenediamine acts as a bridge with two -NH- groups to increase molecular hydrogen bonding, and can bind firmly to multiple proteins. Co-localization of the target compounds **d-Y-B** and **dO-Y-B** with the hoechst dye for labeling living cells showed that these compounds had high biocompatibility and photostability for localization to HeLa cells. *In vivo* imaging in mice can realize specific localization and real-time visualization of tumor cells. The results of cytotoxicity experiments *in vitro* and computer software simulating molecular docking confirmed the potential of the target compounds as an anticancer agents. The bifunctional probe realized visualization of cancer cells in mice, and can kill cancer cells by anti-proliferation, which may provide a direction for future anticancer drug development.

## Introduction

Diagnosis and treatment are two big mountains between human health and anticancer. Targeting cancer cells is the foundation of treatment. However, the existing technical means limit physicians’ ability to monitor cancer cells in real time (Pashayan and Pharoah, 2020; Whitaker, 2020). Optical imaging technology (Zhang et al., 2015; Zhou et al., 2016; Du et al., 2019) based on fluorescent probe is considered as a potential technology for tumor diagnosis by the academic circles because of its advantages of non-invasive and sensitivity (Guo et al., 2018; Karan et al., 2021; Barth et al., 2022). Boron-dipyrromethene (BODIPY) dye (Labra-Vazquez et al., 2020; Xiao et al., 2020; Qiao et al., 2021; Vales et al., 2021; Wen et al., 2021; Wang et al., 2021) has many advantages, such as good light stability (Kyeong et al., 2018; Haldar et al., 2020), large molar extinction coefficient (Che et al., 2020; Pfeifer et al., 2020), stable spectral properties (Zlatic et al., 2020), and narrow spectral half-peak (Zhu et al., 2019; Song et al., 2020). Since it came out, it has attracted the attention of scholars from various circles. In particular, as a widely used biological imaging reagent, the development of organic dyes (including BODIPY) with strong absorption and fluorescence bands in the near infrared (NIR) region has attracted great attention, so that it has the advantages of strong anti-interference ability, and clear and accurate imaging, making it have great potential in the field of optical imaging (Li et al., 2019; Wang et al., 2020; Wang et al., 2021).

Single-function fluorescent probes cannot meet the current research scope in many interdisciplinary disciplines. With the continuous exploration of the application of new fluorescent probes, scientists have gradually turned their attention to the multi-function research field. Chunquan Sheng’s research group designed a series of fluorescent probes based on 10-hydroxyevodiamine (Chen et al., 2019). Using subcellular localization method, it was proved that 10-hydroxyevodiamine could enter mitochondria and lysosomes, and play an anti-tumor role through autophagy. Wu et al. (2017) designed and synthesized a bifunctional probe PPET3-N2 of conjugated poly electrolytes. The probe PEET3-N2 enters lysosomes through endocytosis, so that lysosomes in autophagy cells can be monitored in real time and autophagy inhibitors can be screened. On the other hand, under the irradiation of white light, the probes could generate singlet oxygen, which will lead to cell death.

Inspired by this, at the beginning of the present study, we figured out that the introduction of external active groups could change the biocompatibility of BODIPY and endow the target compound with antitumor activity. Pyrimidine derivatives, an antimetabolite that has been active in clinical practice for more than 60 years, have aroused our interest (Shao et al., 2021; Xu et al., 2021). The 2,4,5, and 6 sites of the pyrimidine ring can be substituted, and the existence of multiple modified sites makes pyrimidine occupy an indispensable position in the field of organic synthesis (De Coen et al., 2016; Xu et al., 2020; Mittersteiner et al., 2021). The wide biological activities of pyrimidine derivatives, such as anti-inflammatory (Shi et al., 2019; Zhao F. et al., 2021; Zhao M.et al., 2021), anti-bacteria (AlNeyadi et al., 2017), anti-tumor (Luo et al., 2018; Deng et al., 2020), and anti-oxidation (Balestri et al., 2018), are also one of the reasons for why pyrimidine is durable. The outstanding anticancer properties of pyrimidine derivatives made us finally decide to connect pyrimidine ring to the target compound as the anti-tumor active group.

Based on this, we designed and synthesized the target compound with BODIPY as the fluorophore and pyrimidine as the therapeutic group, in order to obtain a new compound that retains the ability of BODIPY fluorescent probe and the antitumor activity of pyrimidine. Our experimental results will provide the basis for further research on the bifunctional anticancer agent for the non-destructive detection of tumor cells and the simultaneous treatment of tumor cells.

## Experimental section

### Chemistry

#### General materials

All solvents and reagents were commercially purchased and used without further purification unless otherwise stated. All glassware were oven-dried and kept in a desiccator before use. Thin-layer chromatography (TLC) was used to monitor the progress of the reaction. TLC results were analyzed at 254 nm and 360 nm under ultraviolet lamp. The reaction products were isolated and purified by column chromatography (silica gel 200–230 mesh). Characterization of the products at each stage by NMR spectroscopy, ^1^H, and ^13^C NMR spectra were recorded on Bruker Avance II spectrometer in CDCl_3_ (400 MHz for ^1^H and 100 MHz for ^13^C); chemical shifts are expressed in ppm, versus internal tetramethyl silane (TMS) = 0 for ^1^H and ^13^C. Coupling constants (*J*) are given in Hz. The molecular weight by MS-ESI (Jeol Ltd. JMS-HX 110/110A) was performed.

### General procedure for the synthesis of d-Y-BP and dO-Y-BP

Using the previous product **B** (1.33 mmol) of our research group as the raw material, triethylamine (2.0 mmol) as the base was reacted with ethyl 4-chloro-2-(methyl thio) pyrrolidine-5-carboxylate (1.33 mmol) in dichloromethane at room temperature for 20 h. The reaction was monitored by TLC, the solvent was removed by evaporation by rotation, and the products were eluted on silica gel column with ethyl acetate and petroleum ether (1:10). Red solid **Y-BP** was finally obtained with a yield of 85%.

**Y-BP** (1.05 mmol) was heated with excess hydrazine hydrate in methanol for 24 h. After the solvent was removed by evaporation, the product was purified on a silica gel column eluting with ethyl acetate in a ratio of 1:4 to petroleum ether. Finally, 700 mg of reddish-brown solid **d-Y-BP** was obtained in a yield of 61%. **d-Y-BP** (0.10 mmol) was oxidized in dichloromethane at room temperature by the oxidizing agent m-Chloroperbenzoic acid (m-CPBA). The reaction was monitored by TLC, and the reaction was completed within 40 min. After the solvent was removed by rotary evaporation, the product was purified on a silica gel column and eluted with ethyl acetate in a ratio of 1:1 to petroleum ether. Finally, a reddish-brown solid **dO-Y-BP** was obtained in a yield of 8.4%.

### Spectral data of synthetic compounds d-Y-BP and dO-Y-BP

4-((2-((8-ethyl-5,5-difluoro-7,9-dimethyl-10-phenyl-5H-5λ^4^,6λ^4^-dipyrrolo [1,2-c:2′,1′-f][1,3,2]diazaborinin-2-yl)amino)ethyl)amino)-N′-(4-((2-((8-ethyl-5,5-difluoro-7,9-dimethyl-10-phenyl-5H-5λ^4^,6λ^4^-dipyrrolo [1,2-c:2′,1′-f][1,3,2]diazaborinin-3-yl)amino)ethyl)amino)-2-(methylthio)pyrimidine-5-carbonyl)-2-(methylthio)pyrimidine-5-carbohydrazide (**d-Y-BP**).

Carmine solid; Yield: 16%; ^1^H-NMR: (400 MHz, CDCl_3_, ppm): *δ*8.70 (t, *J* = 6.0 Hz, 2H),8.28 (s, 2H), 7.44–7.28 (m, 10H), 6.47 (d, *J* = 4.0 Hz, 2H), 6.01 (d, *J* = 8.0 Hz, 2H), 3.81 (d, *J* = 4.0 Hz, 4H), 3.59 (d, *J* = 4.0 Hz, 4H), 2.51 (s, 6H), 2.45 (s, 6H), 2.32–2.29 (m, 4H), 1.37 (s, 6H); ^13^C NMR (100 MHz, CDCl_3_, ppm): *δ*175.02, 167.65, 160.11, 159.67, 153.48, 145.31, 135.25, 133.57, 133.06, 132.61, 132.33, 130.14, 129.69, 128.40, 128.03, 105.84, 102.95, 43.76, 40.09, 17.15, 14.94, 14.08, 11.94, and 11.56. MS (ESI) *m/z* calculated for C_54_H_58_B_2_N_14_O_2_S_2_ [M+3H_2_O+H]^+^1151.4826, found 1151.6417.

4-((2-((8-ethyl-5,5-difluoro-7,9-dimethyl-10-phenyl-5H-5λ^4^,6λ^4^-dipyrrolo [1,2-c:2′,1′-f][1,3,2]diazaborinin-2-yl)amino)ethyl)amino)-N′-(4-((2-((8-ethyl-5,5-difluoro-7,9-dimethyl-10-phenyl-5H-5λ^4^,6λ^4^-dipyrrolo [1,2-c:2′,1′-f][1,3,2]diazaborinin-3-yl)amino)ethyl)amino)-2-(methylsulfonyl)pyrimidine-5-carbonyl)-2-(methylthio)pyrimidine-5-carbohydrazide (**dO-Y-BP**).

Carmine solid; Yield: 45%; ^1^H-NMR: (400 MHz, CDCl_3_, ppm): *δ*8.41 (s, 2H), 7.48–7.30 (m, 12H), 6.42 (d, *J* = 4.0 Hz, 2H), 6.14 (s, 2H), 3.76 (s, 4H), 3.62 (s, 4H), 3.33 (s, 6H), 2.41 (s, 6H), 2.00 (d, *J* = 4.0 Hz, 4H), 1.37 (s, 6H), 1.30 (s, 6H), 1.00 (s, 6H); ^13^C NMR (100 MHz, CDCl_3_, ppm): δ 178.45, 135.38, 135.36, 133.07, 132.44, 132.30, 131.91, 130.57, 129.53, 129.40, 129.07, 128.18, 128.06, 127.85, 30.41, 29.38, 19.29, 16.57, 14.07, 10.61, 10.34. MS (ESI) m/z calculated for C_54_H_58_B_2_N_14_O_6_S_2_ [M+6H_2_O+K+CH_3_CN]^+^1348.4764, found 1348.4302.

### Spectroscopic properties

Ultraviolet-visible (UV–vis) spectra were recorded on a UV-2550 spectrophotometer using a 1 cm path length quartz cuvette and fluorescence Spectra were performed on Shimadzu RF-5301PCS spectrofluorophotometer at room temperature. A proper amount of the compound was dissolved in DMSO and prepared into 1 mM mother liquor for later use. Spectral tests of solutions with different concentrations were prepared according to needs and data were recorded. The UV-Vis wavelength range is 400–700 nm. The fluorescence of the compounds were obtained at the optical path of 10 mm and the excitation wavelength of 500 nm, and the wavelength range of the recorded emission was 500–700 nm.

Considering the solubility of the compound and the biological toxicity of the solvent, DMSO/water = 1:9, V/V was selected as the solvent to determine the stability of the fluorescence intensity of the three compounds in the solvent within 1 and 5 min. In addition, PBS buffer was used to prepare solutions with pH values from 7.5 to 5.5, and DMSO/buffer = 1:9, V/V was selected as the solvent to test the stability of the compounds in solutions with different pH values, using DMSO/water = 1:9, V/V as the solvent as the control.

### Biological

#### Cell culture and treatment

The cytotoxic activities of the compounds were screened against two human tumor cell lines and human normal liver *in vitro*. The cell lines HCT-116 (human colon cancer cell line), HeLa (human cervical cancer cell line), and L-02 (human normal liver cell line) were all from the American type culture collection. L-02 were cultured in DMEM, while HCT-116 and HeLa were routinely cultured in RPMI-1640. All cell lines were supplemented with 10% fetal bovine serum and adams fusion method in a humidified atmosphere of CO_2_/air (5/95%) at 37°C. Cancer and normal cells were monitored daily and kept at 80% cell density.

### Cell imaging

HeLa in the logarithmic phase was treated with cellular trypsin, which was then inoculated on 96-well plates and cultured in CO2/AIR (5/95%) AT37C for 24 h. Dissolve the compound in DMSO to give the appropriate concentration. The original culture solution of cells in each well was removed, replaced with a medium containing 10 μg/ml of different samples, and treated for 48 h. The culture medium was removed, washed with PBS for two times and then treated with paraformaldehyde stationary liquid for 25 min. The fixative was removed, washed twice with PBS and stained with DAPI for 25 min. Finally, the staining solution was discarded, washed twice with PBS, and treated with anti-fluorescence quenching scaffold. The fluorescence image of cells was obtained under fluorescence microscope.

After the Hela grew for 24 h, the original culture medium of HeLa was removed, washed once with PBS, and then 2 ml of diluted samples were added to the confocal dish, and the culture continued for 48 h. After drug treatment for 48 h, add appropriate hoechst, remove the cell culture solution, and add 500 μl diluted dye solution to each dish. Incubate in an incubator at 37°C for 10–15 min, observe the dyeing effect with fluorescence microscope, and take photos with confocal microscope at appropriate wavelength.

### *In vitro* cytotoxicity

The cytotoxicity of cancer cells and normal cells in the logarithmic phase was monitored by the MTT assay. All cells were seeded on 96 well plates at a rate of 106 cells/well. The three cell lines were treated with different concentrations of the compound and the control 5-FU. After 24 h, the supernatant was dissolved in DMSO and the optical density of the samples was measured by microplate photometer at 490 nm. Cell viability was expressed as percent change in absorbance relative to control values.

### *In vivo* imaging

The compounds were same concentrations (10 μM, 10 μl in DMSO/saline = 1:9, V/V) were injected in an 8-week-old mouse weighing 21 g. The fluorescence intensity of subcutaneous tumor tissue was detected 30 min later.

### Molecular docking

To understand the interaction of compounds with receptors at the atomic level, thymidylate synthase crystals were downloaded from Protein database (PDB ID: 6QYQ) for molecular docking with the target compounds. Molecular docking was performed to evaluate 2D and 3D binding patterns of compounds at enzyme active sites. AutoDock Vina 1.1.2. software was used for docking, and pymol 1.5.6 was used for processing and analyzing the docking results.

## Results and discussion

### Chemistry

**SCHEME 1:**
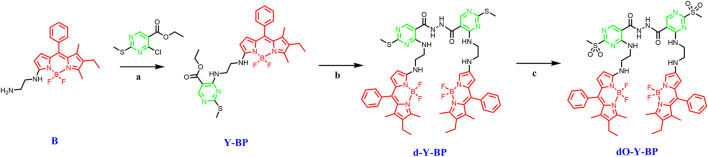
The synthetic routes of **d/dO-Y-BP**. Reagents and conditions: (a) TEA (1.5 equiv), DCM, r.t.; (b) hydrazine hydrate (1.5 equiv), CH_3_OH, r.t.; (c) m-Chloroperbenzoic acid (1.5 equiv), DCM, r.t.

The synthesis route of the target compound is shown in the Scheme 1. First, ethylenediamine is introduced into the parent nucleus of BODIPY, and the intermediate Y-BP is obtained by connecting it to 4-chloro-2-(methyl thio-pyrrolidine)-5-carboxylic acid ethyl ester at room temperature. Then Y-BP and hydrazide hydrate are reflux in methanol to obtain the target compound d-Y-BP with bis-amide as its symmetry axis. The two methyl thiols of D-Y-BP were oxidized under the action of the m-Chloroperbenzoic acid (m-CPBA) to obtain the product containing methyl sulfonyl Do-Y-BP. The whole reaction process was monitored by TLC and the target product was obtained by silica gel column chromatography.

Using ethylenediamine as a bridge, a fluorescent probe drug with anticancer activity was obtained by combining BODIPY with pyrimidine. Not only that, ethylenediamine two -NH- groups to increase intermolecular hydrogen bonding ability, and can firmly bind with a variety of proteins.

### Spectroscopic properties

**FIGURE 1:**
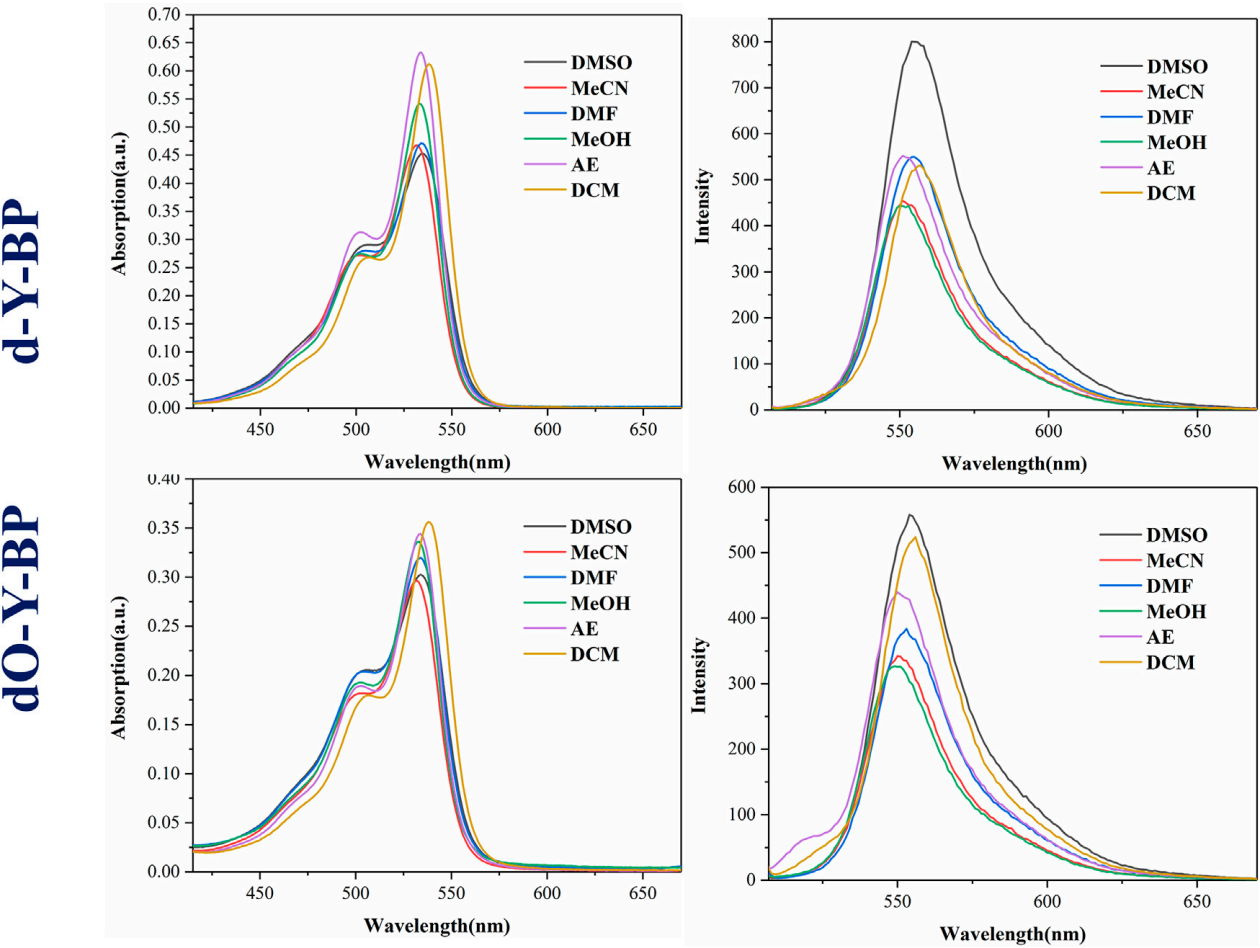
Effect of different solvents on photophysical properties of **d/dO-Y-BP**.

The absorption spectra and emission spectra of target compounds **d-Y-BP** and **dO-Y-BP** in DMSO, MeCN, DMF, MeOH, AE, and DCM are shown in Figure 1, and the detailed data are recorded in Table 1. The concentration of d-Y- BP in the different solvents is 5.1 μM and that of **dO-Y-BP** is 4.6 μM. The maximum absorption peaks of the two compounds are concentrated in the range of 532–538 nm, in which **d-Y-BP** shows the maximum ultraviolet absorption in AE and DCM with low polarity, and a minimum ultraviolet absorption in DMSO with high polarity. The ultraviolet absorption values of **dO-Y-BP** in different solvents had no obvious difference. In addition, the existence of the dipole moment caused a slight shift of the maximum absorption peak of the compound in solvents with different polarities.

**TABLE 1:** Photophysical characteristic of **d/dO-Y-BP** in different solvents.

Compound	Solvents	λ_abs max_, nm/Abs.,a.u	λ_em max_, nm/FL intensity	Δ_max_ν, cm^−1^	Φ_f_
d-Y-BP	DMSO	535/0.453	554/800	641	0.341
	MeCN	532/0.468	551/455	648	0.109
	DMF	534/0.471	555/549	709	0.209
	MeOH	533/0.542	550/443	580	0.112
	AE	534/0.633	551/552	578	0.136
	DCM	538/0.612	557/531	634	0.149
dO-Y-BP	DMSO	534/0.302	554/558	676	0.290
	MeCN	32/0.296	550/341	615	0.140
	DMF	534/0.320	553/384	643	0.174
	MeOH	533/0.336	549/327	547	0.127
	AE	534/0.344	550/440	545	0.198
	DCM	538/0.356	556/524	602	0.201

The maximum emission wavelengths of **d-Y-BP** and **dO-Y-BP** are between 500–557 nm, and the maximum emission wavelengths of the compounds in different solvents are very similar. The fluorescence intensity of **d-Y-BP** with the same concentration in DMSO was 1.8 times higher than that in methanol, but the fluorescence intensity of **d-Y-BP** in other solvents has no difference. The fluorescence intensity of **dO-Y-BP** in DMSO and DCM was significantly higher than that of the other four solvents. Stokes shift of different compounds in different solvents are greater than or equal to 550 cm^−1^, which has no obvious relation with the polarity of the solvent.

Using Rhodamine B (Φ_f_ = 0.7) as the standard substance, the fluorescence quantum yields of compounds in different solvents were determined. In DMSO, both **d-Y-BP** and **dO-Y-BP** showed high fluorescence quantum yields.

Generally, the absorption and emission spectra of the target compounds are hardly affected by the change of solvent polarity. The results of the spectral data show that **d-Y-BP** and **dO-Y-BP** had good photophysical properties and fluorescence emission at μM concentration. So that two compound can be used for cell image.

### Stability study

**FIGURE 2:**
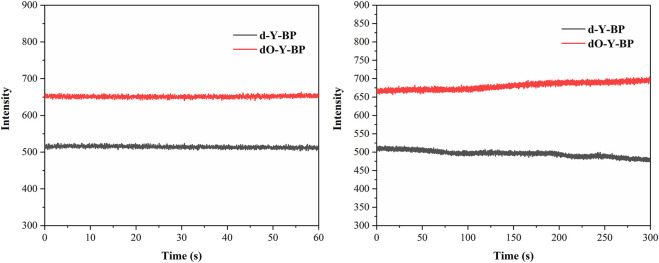
Fluorescence stability of **d/dO-Y-BP**.

Considering the solubility of the compound and the cytotoxicity of the solvent, DMSO was selected as the solvent to determine the stability of the target compound for 1 and 5 min using the maximum ultraviolet absorption wavelength of the compound in DMSO as the excitation wavelength and the maximum fluorescence emission wavelength as the emission wavelength (Figure 2). **d-Y-BP** and **dO-Y-BP** show excellent stability within 1 min and slight fluctuation stability within 5 min.

In addition, DMSO/water = 1:9, v/v was used to prepare solutions with different pH values, so as to determine the stability of the target compound at different pH values of 1, 10, 20, 30, and 40 min. The results are displayed in the supporting information. The stability of these two compounds at different pH levels was very good at 1 min. The stability of d-Y- BP was relatively stable at 10 min, and the fluorescence intensities decreased to varying degrees at 20, 30, and 40 min, which indicated that the stability of **d-Y-BP** was barely acceptable. The fluorescence intensity of **dO-Y-BP** fluctuated greatly in different pH solutions and showed a downward trend as a whole. The oxidation of methylthio to methanesulfonyl fluctuates the fluorescence intensity of **dO-Y-BP**.

### Cell imaging

**FIGURE 3:**
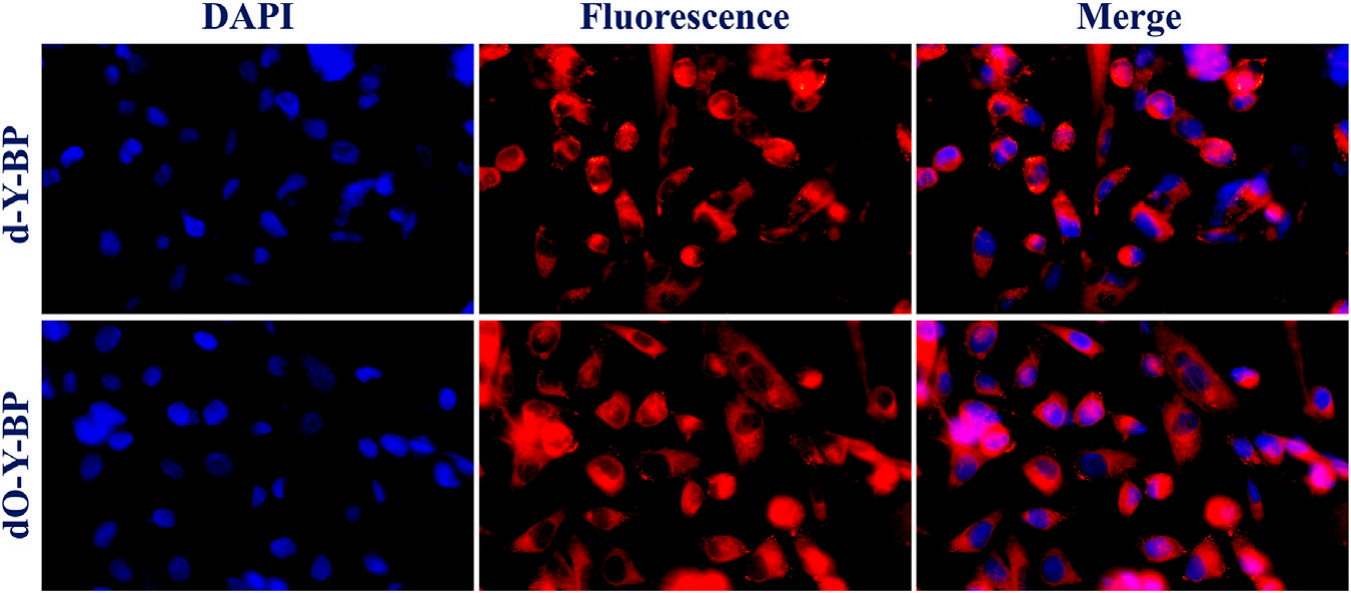
Cell imaging of treated HeLa cells with **d/dO-Y-BP** for 48 h.

In order to understand the possibility of applying target compound in cancer diagnosis and treatment, we provided the HeLa cells incubated with DAPI for 25 min after 48 h of co-incubation with the target compound. Fluorescence images taken by low-resolution fluorescence microscope show that the target compounds are distributed around the cytoplasm and nucleus, and pass through the cell membrane and emit remarkable fluorescence (Figure 3).

**FIGURE 4:**
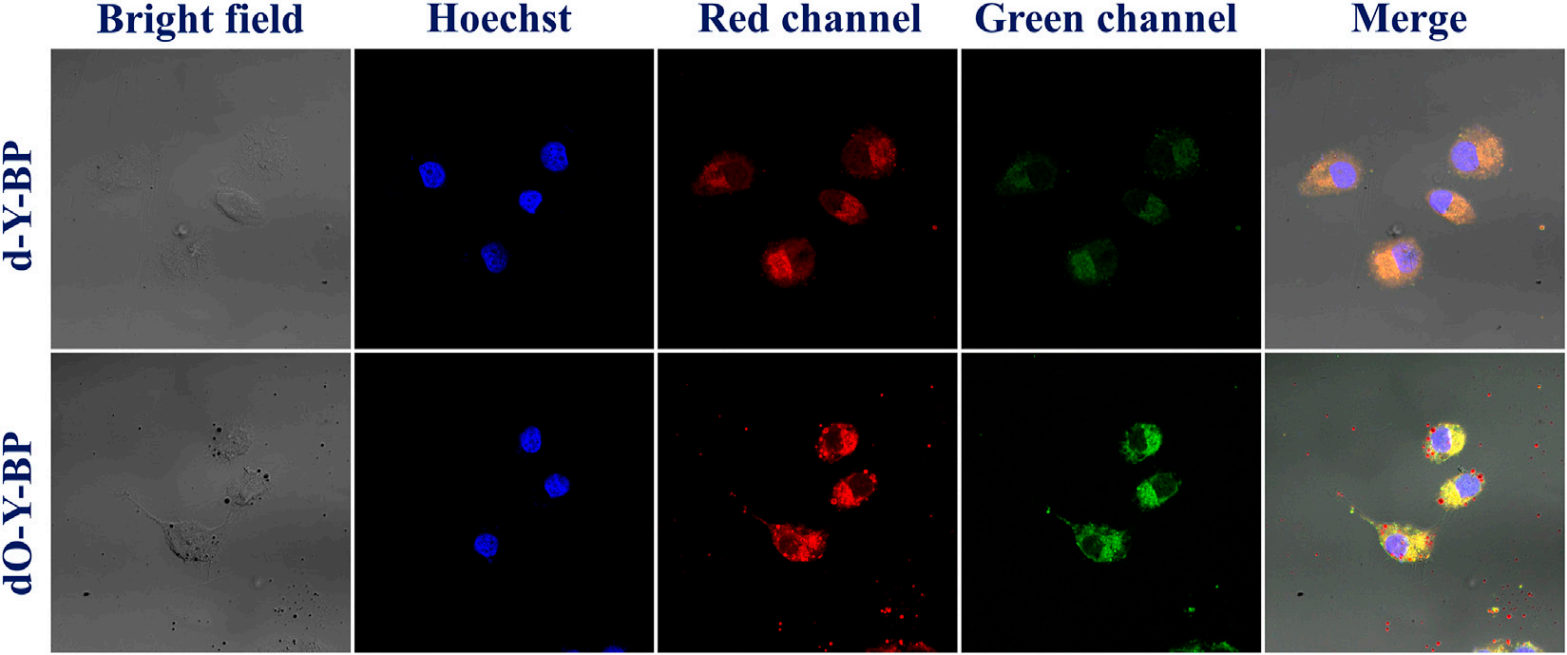
Fluorescent microscope imaging of treated HeLa cells with **d/dO-Y-BP** for 48 h.

HeLa cells were cultured in the medium containing 10 μg/ml of the target compound for 48 h and then co-cultured with hoechst dye for 10–15 min to obtain images were obtained under a confocal microscope. HeLa cells treated with **d-Y-BP** and **dO-Y-BP** showed bright and visible red or green fluorescence in different channels, which indicated that the target compound could enter the cytoplasm through the cell membrane, and even a small number of the compound could enter the nucleus (Figure 4). Cell imaging also showed that these two compounds can meet the requirements of bright signals of fluorescent dyes in cells after entering cancer cells. The fluorescent brightness of **dO-Y-BP** was even higher than that of Hoechst dye. The good picture quality indicates that the target compounds **d-Y-BP** and **dO-Y-BP** have good biocompatibility and the ability to label cancer cells.

### *In vitro* cytotoxicity

**FIGURE 5:**
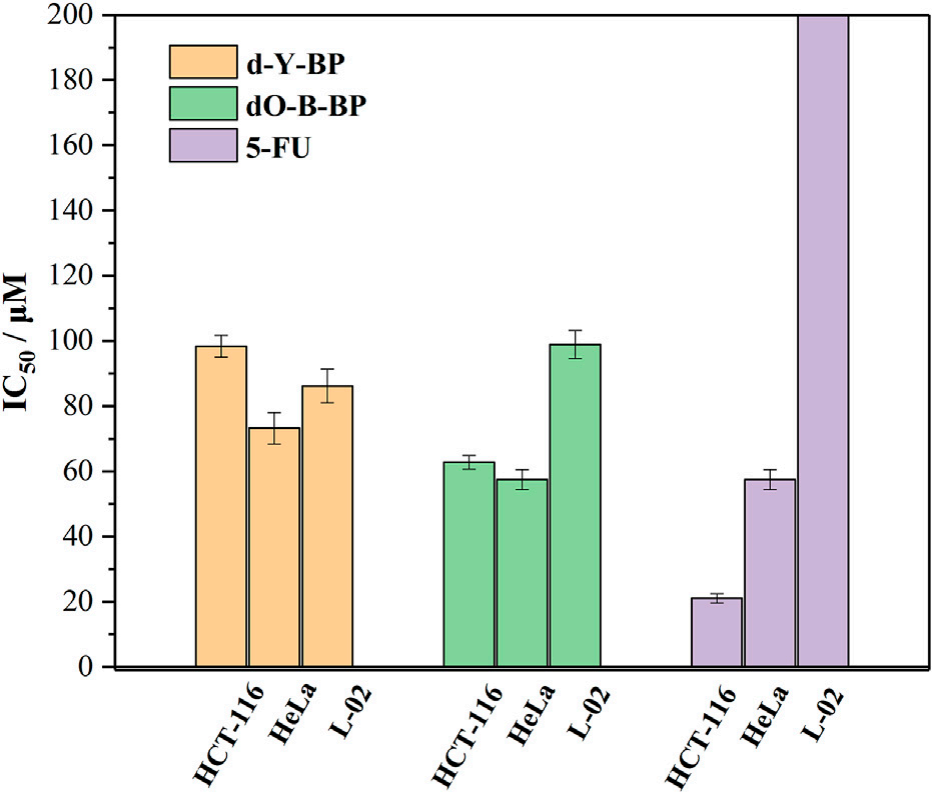
The half-maximum inhibitory concentration (IC_50_) of **d/dO-Y-BP** and 5-FU on HCT-116, HeLa, and L-02 cells.

With 5-FU as a positive control, the anti-proliferation activity of that target compounds **d-Y-BP** and **dO-Y-BP** on human colon cancer cell line (HCT-116), human cervical cancer cell line (HeLa), and human normal liver cell line (L-02) were detected by MTT assay, and the result are shown in the figure. The IC_50_ values of **d-Y-BP** for these three cell lines were 98.33 ± 3.32, 73.22 ± 4.79, and 86.15 ± 5.14 μM, respectively. **d-Y-BP** showed inhibitory activity on cancer cells and normal cells, with the strongest inhibition on HeLa cells. The IC_50_ values of **dO-Y-BP** were 62.79 ± 2.09, 57.47 ± 3.02, and 98.88 ± 4.34 μM, respectively. Compared to **d-Y-BP**, **dO-Y-BP** shows selective inhibition of cancer cell activity. **dO-Y-BP** has a strong inhibitory effect on HeLa cells, which was 1.7 times that of normal cells. The structure-activity relationship of the compounds was studied, and it was found that the oxidation of sulfur atoms attached to pyrimidine ring could significantly reduce the cytotoxicity of the compound and enhance their anti-proliferation activity on cancer cells (Table 2; Figure 5).

**TABLE 2:** The half-maximum inhibitory concentration (IC_50_) of **d/dO-Y-BP** and 5-FU on HCT-116, HeLa, and L-02 cells.

IC_50_ (μM) ± SD^a^			
Compound	HCT-116	HeLa	L-02
d-Y-BP	98.33 ± 3.32	73.22 ± 4.79	86.15 ± 5.14
dO-Y-BP	62.79 ± 2.09	57.47 ± 3.02	98.88 ± 4.34
5-FU	21.03 ± 1.39	18.07 ± 3.11	>200

a Data are means ± SD, of triplicate experiments.

The results of *in vitro* cytotoxic activity indicated that the compound **dO-Y-BP**, as an anticancer compound, was worthy of further study.

### In vivo imaging

**FIGURE 6:**
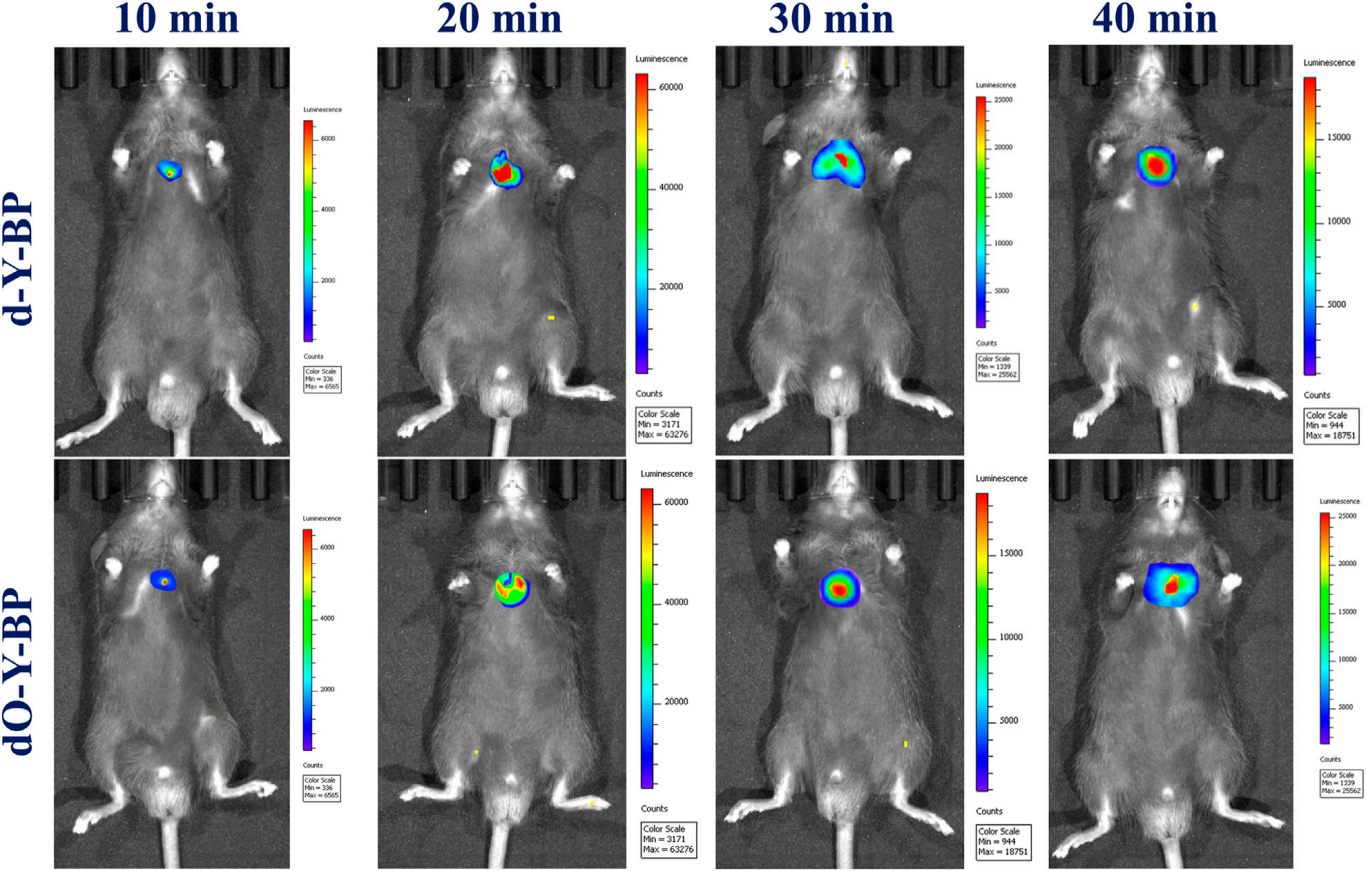
*In vivo* imaging of changes in the levels of **d/dO-Y-BP** in mice lung tumor tissues.

The target compounds **d-Y-BP** and **dO-Y-BP** (10 μM, 10 μl in DMSO/Saline = 1: 9, v/v) were subcutaneously injected into aged 21 g male mice at 8 weeks, and the fluorescence intensities of the subcutaneous tumor tissues was observed. As shown *in vivo* imaging results of mice, after 10 min of subcutaneous injection, obvious fluorescence was observed under the skin of mice, and the fluorescence intensities of the two compounds reached its peak at 20 min. In the next 20 min, the fluorescence intensity of the subcutaneous tumor tissue decreased slightly, but the fluorescence was still strong. The above results indicate that the target compounds **d-Y-BP** and **dO-Y-BP** can rapidly target the mouse tumor tissues and continuously emit bright fluorescent signals. This provided data support for the application of the target compounds in the field of tumor diagnosis (Figure 6).

### Molecular docking study

**FIGURE 7:**
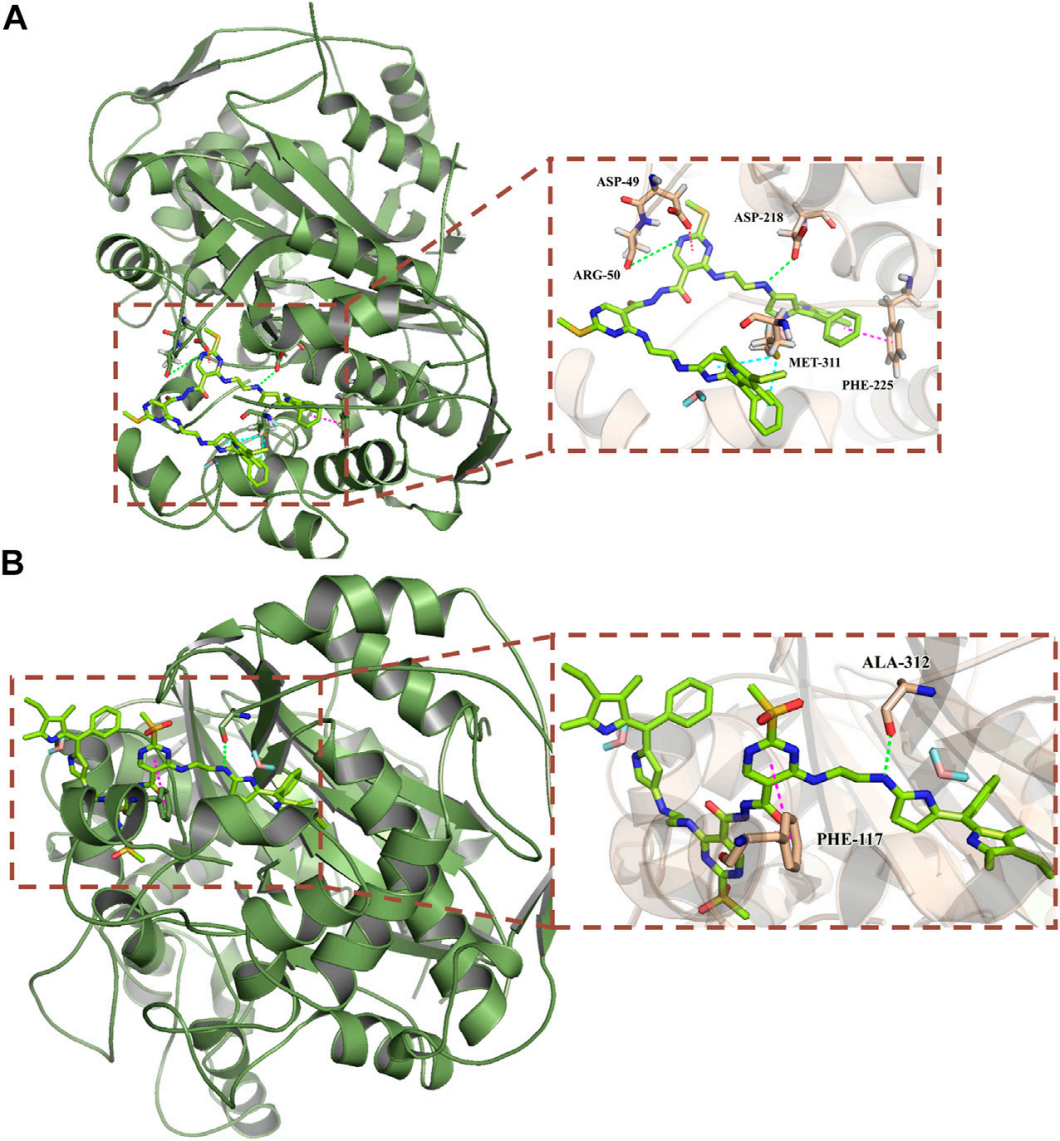
Predicted binding mode of compounds **d-Y-BP (A)** and **dO-Y-BP (B)** at the binding site of thymidylate synthase (PDB ID: 6QYQ).

Thymidine synthase is an essential enzyme necessary for DNA synthesis. 5-FU can reduce the DNA replication of cancer cells by inhibiting the activity of thymidine synthase, so as to achieve the anti-cancer effects. In order to further understand the anticancer mechanism of the target compounds **d-Y-BP** and **dO-Y-BP**, thymidylate synthase (PDB ID: 6QYQ) was selected for molecular docking with the target compound. **d-Y-BP** and **dO-Y-BP** docking scores were 151.32 and 118.879, respectively. The results of docking are shown in Figure 7A. The amino acid residue ARG-50 oxo formed a hydrogen bond with the NH group on the **d-Y-BP** pyrimidine ring, and at the same time, the pyrimidine ring also has π-anion interaction with the ASP-49 residue. The carbonyl oxygen of ASP-218 also forms hydrogen bond with that NH group of ethylenediamine. And benzene ring in Pyrrole **d-Y-BP** group reacts with MET-311 residue at the same time to produce π-sulfur effect. The benzene ring in the symmetric group and PHE-225 strengthened their relationship through π-π stacking. Similar to the scoring results, **dO-Y-BP** scored lower than **d-Y-BP** with less linkage to amino acid residues in its interactions with amino acid residues (Figure 7B). Only the pyrimidine ring and the benzene ring at PHE-117 residue form π-π stacking, and the carbonyl oxygen of ALA-312 and NH group of ethylenediamine form hydrogen bonds.

The results of molecular docking showed that d-Y-BP more superior biological activity than **dO-Y-BP**, and **d-Y-BP** had a better effect on thymidylate synthase. We provide additional interaction of the target compound with thymidylate synthase in the supporting information.

## Conclusion

In summary, based on two groups of fluorescent probes with different functional groups, we developed a kind of fluorescent probes with anti-tumor activity. Through amination and oxidation reaction, we obtained probes **d-Y-B** and **dO-Y-B** with high biocompatibility, which were used for *in vivo* visualization of cancer cells and anti-tumor. **d-Y-B** and **dO-Y-B** retained the advantages of BODIPY as a fluorescent dye, and they could produce clear and bright images *in vivo* or *in vitro*, which can be used for precise location of cancer cells. As anticancer agents, these two compounds show good affinity for thymidylate synthase, and oxidized **dO-Y-B** showed better cytotoxicity. The successful synthesis of two fluorescent probes with excellent biocompatibility and good optical properties provides a new idea and method for the further development of fluorescent antitumor drugs.

## Data Availability

The original contributions presented in the study are included in the article/Supplementary Material, further inquiries can be directed to the corresponding author.
