# Supplementary material for: Symmetry dual functional pyrimidine-BODIPY probes for imaging targeting and activity study
Source: Front Chem. 2022 Sep 20;10:977008. doi: 10.3389/fchem.2022.977008 (PMC9530934; doi:10.3389/fchem.2022.977008)
Supplement: Supplementary file 1 [file DataSheet1.docx]

**Supporting Information**

**Symmetry dual functional pyrimidine- BODIPY probes for imaging targeting and activity study**

Shuping Xu^1,#^, Ying Liu^2,#^, Zhou Wang^3^, Aolin He^4,^*, Guofan Jin^2,^*

^1^The People’s Hospital of Danyang, Affiliated Danyang Hospital of Nantong University, Zhenjiang 212300, P.R. China

^2^School of Pharmacy, Jiangsu University, Zhenjiang 212013, P.R. China

^3^College of Vanadium and Titanium, Panzhihua University, 617000, P.R. China

^4^Affiliated Kunshan Hospital, Jiangsu University, Suzhou 215300, P.R. China

*****Correspondence: [organicboron@ujs.edu.cn](mailto:organicboron@ujs.edu.cn)；

**Table of Contents**

**Figure S1** **……………………………………………**^1^H NMR of **d-Y-BP**

**Figure S2 ……………………………………………**^13^C NMR of **d-Y-BP**

**Figure S3** **……………………………………………**MS-ESI of **d-Y-BP**

**Figure S4** **……………………………………………**^1^H NMR of **dO-Y-BP**

**Figure S5 ……………………………………………**^13^C NMR of **dO-Y-BP**

**Figure S6** **……………………………………………**MS-ESI of **dO-Y-BP**

**Figure S7** **……………………………………………**^1^H NMR of **Y-BP**

**Figure S8 ……………………………………………**^13^C NMR of **Y-BP**

**Figure S9** **……………………………………………**MS-ESI of **Y-BP**

**Figure S10** **…………………………………………**UV-Vis absorption spectrum of **dO-Y-BP**

**Figure S11 …………………………………………**UV-Vis absorption spectrum of **dO-Y-BP**

**Figure S12** **…………………………………………**Fluorescence spectrum analysis of **d-Y-BP**

**Figure S13 ……………………………………….**Fluorescence spectrum analysis of **dO-Y-BP**

**Figure S14** **…………………………………………**Stability of **d-Y-BP**

**Figure S15** **…………………………………………**Stability of **dO-Y-BP**

Figure S1. ^1^H NMR of **d-Y-BP**

Figure S2. ^13^C NMR of **d-Y-BP**

Figure S3. MS-ESI of **d-Y-BP**

Figure S4. ^1^H NMR of **dO-Y-BP**

Figure S5. ^13^C NMR of **dO-Y-BP**

Figure S6. MS-ESI of **dO-Y-BP**

Figure S7. ^1^H NMR of **Y-BP**

Figure S8. ^13^C NMR of **Y-BP**

Figure S9. MS-ESI of **Y-BP**

Figure S10. UV-Vis absorption spectrum of **d-Y-BP**

Figure S11. UV-Vis absorption spectrum of **dO-Y-BP**

Figure S12. Fluorescence spectrum analysis of **d-Y-BP**

Figure S13. Fluorescence spectrum analysis of **dO-Y-BP**

Figure S14. Stability of **d-Y-BP**

Figure S15. Stability of **dO-Y-BP**
